# Supplementary material for: Biowaste-Derived, Highly Efficient, Reusable Carbon Nanospheres for Speedy Removal of Organic Dyes from Aqueous Solutions
Source: Molecules. 2022 Oct 18;27(20):7017. doi: 10.3390/molecules27207017 (PMC9610970; doi:10.3390/molecules27207017)
Supplement: Supplementary file 1 [file molecules-27-07017-s001.zip › molecules-1971975-supplementary.pdf]

Supplementary Information

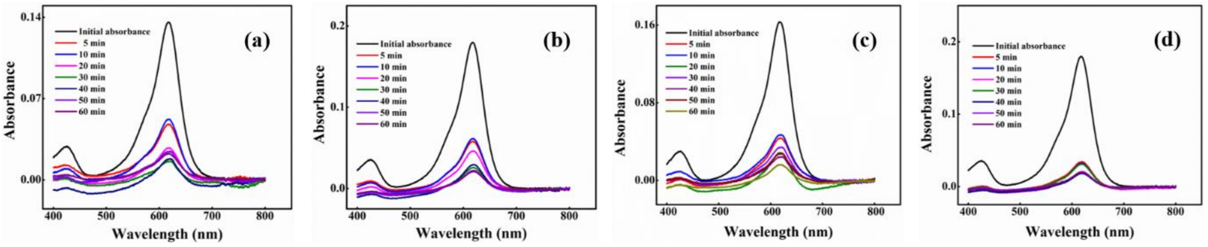

Figure S1. UV-Vis spectra for adsorption of MG when CNSs of different carbonization temperature were used (a) 400 °C (b) 600 °C (c) 800 °C (d) 1000 °C

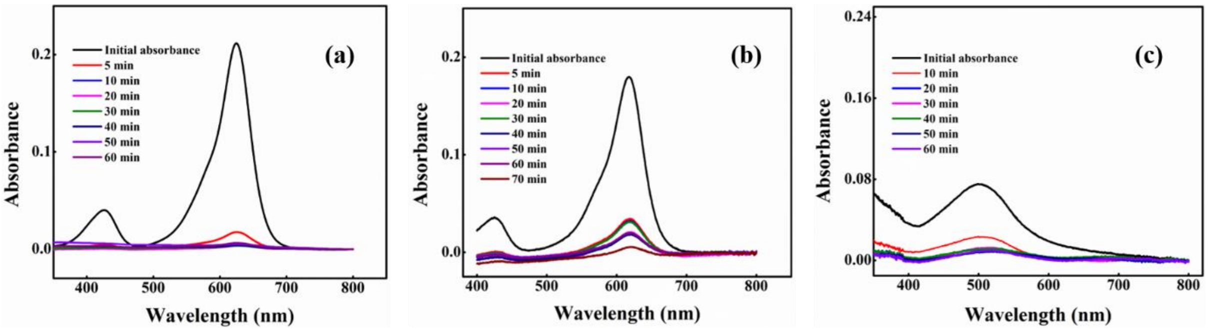

Figure S2. UV-Vis spectra for adsorption of dyes as function of contact time (a) BG (b) MG (c) CR

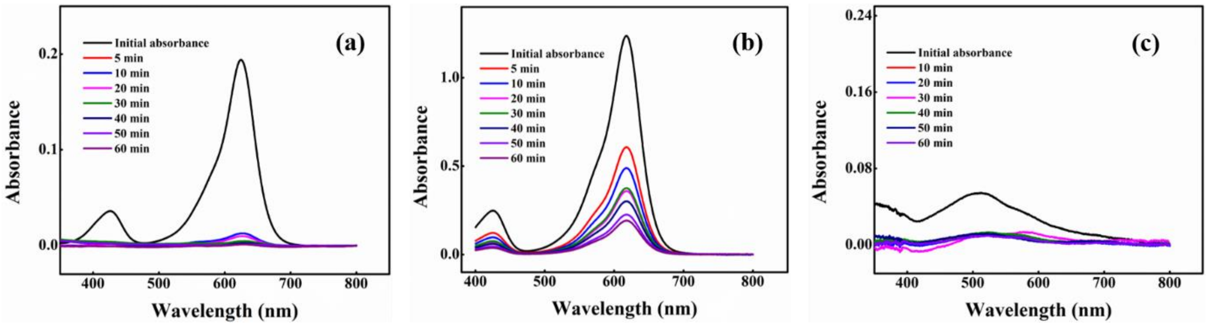

Figure S3. UV-Vis spectra for adsorption of dyes as function of contact time when 0.5 mg of adsorbent dosage was taken (a) BG (b) MG (c) CR

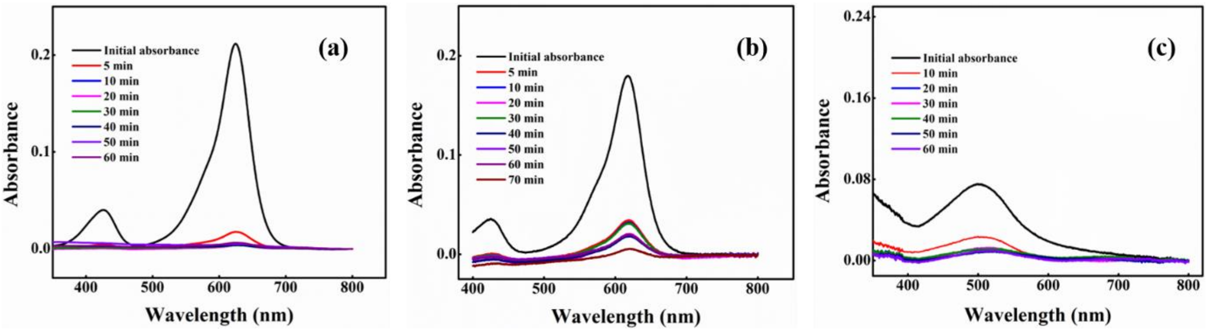

Figure S4. UV-Vis spectra for adsorption of dyes as function of contact time when 1 mg of adsorbent dosage was taken (a) BG (b) MG (c) CR

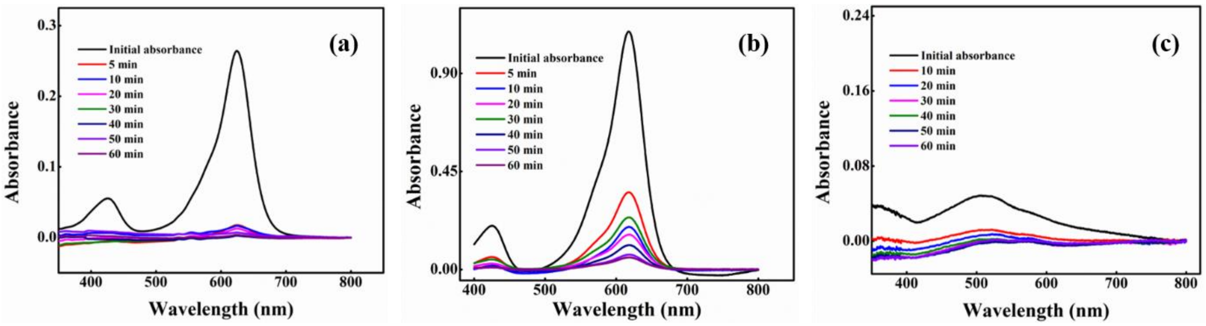

Figure S5. UV-Vis spectra for adsorption of dyes as function of contact time when 1.5 mg of adsorbent dosage was taken (a) BG (b) MG (c) CR

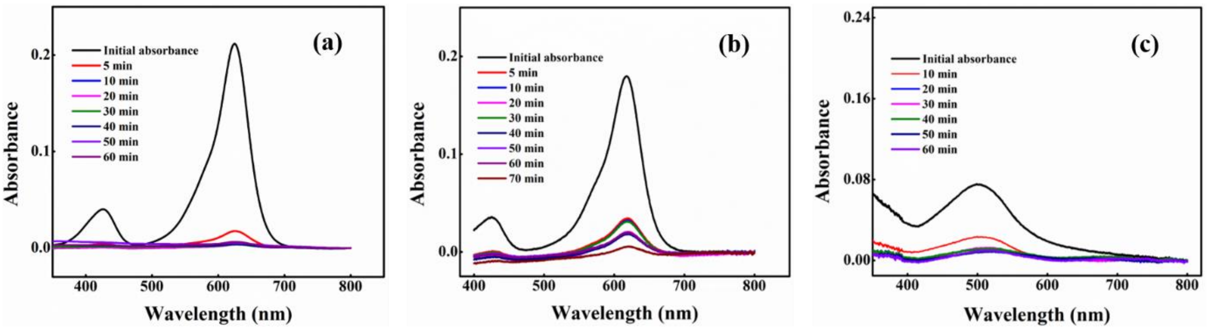

Figure S6. UV-Vis spectra for adsorption of dyes as function of contact time when 10 μM initial concentration was considered (a) BG (b) MG (c) CR

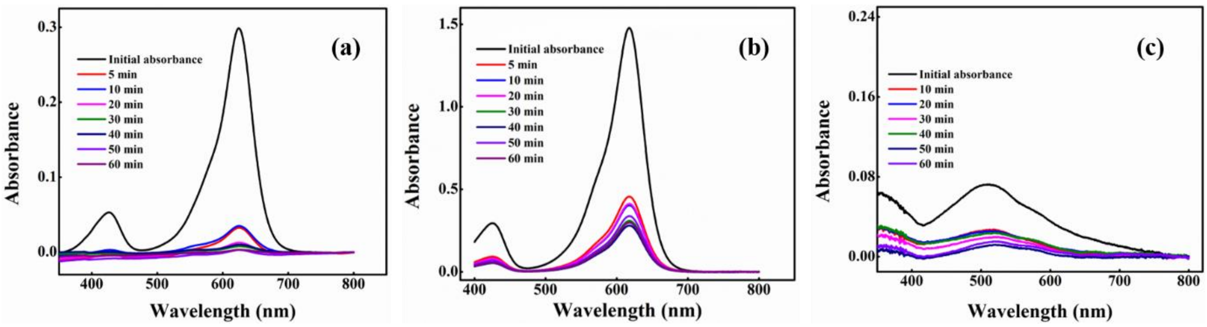

Figure S7. UV-Vis spectra for adsorption of dyes as function of contact time when 15 μM initial concentration was considered (a) BG (b) MG (c) CR

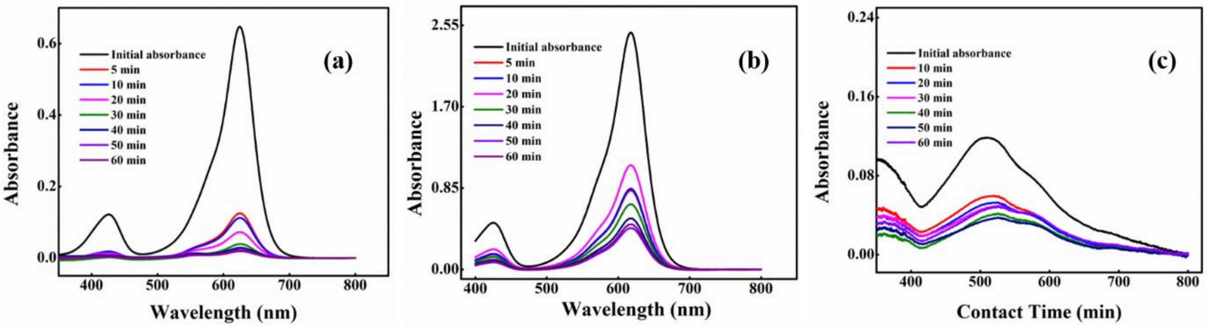

Figure S8. UV-Vis spectra for adsorption of dyes as function of contact time when 20 μM initial concentration was considered (a) BG (b) MG (c) CR

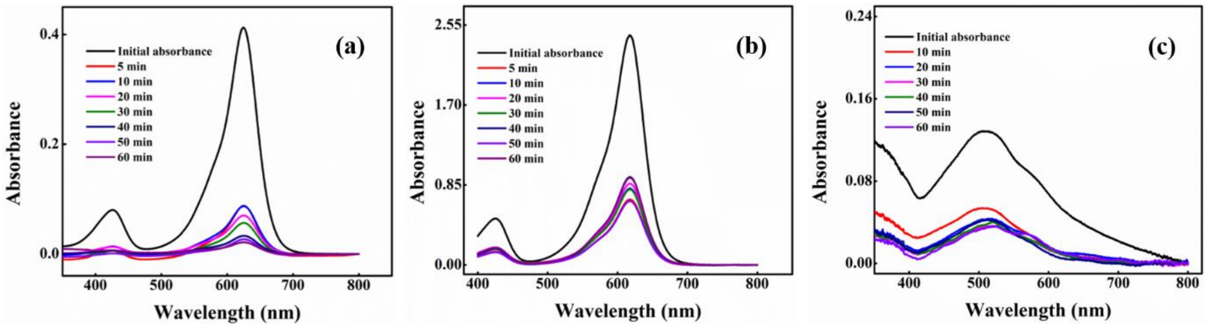

Figure S9. UV-Vis spectra for adsorption of dyes as function of contact time when 25 μM initial concentration was considered (a) BG (b) MG (c) CR

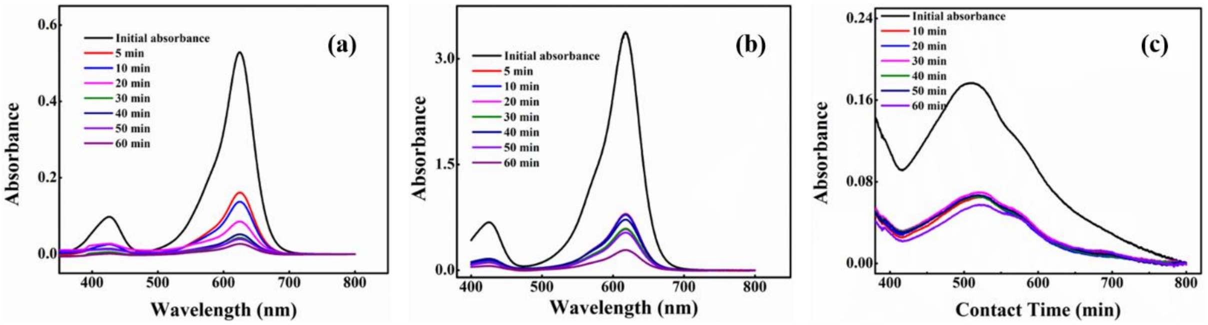

Figure S10. UV-Vis spectra for adsorption of dyes as function of contact time when 30 μM initial concentration was considered (a) BG (b) MG (c) CR

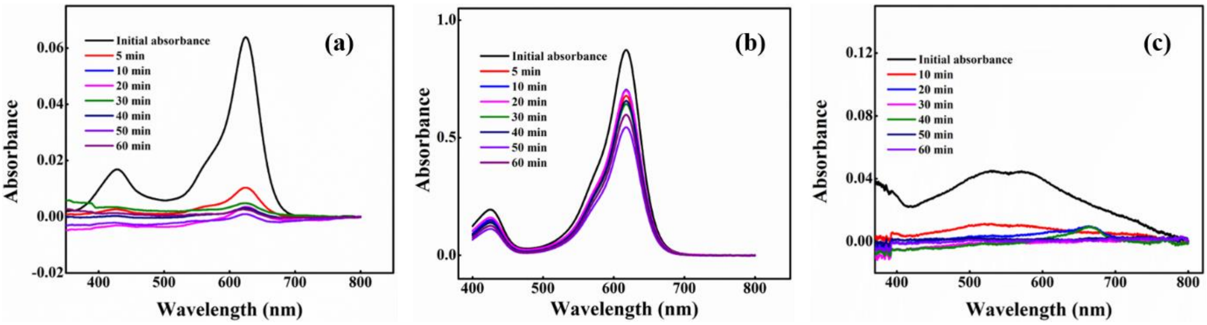

Figure S11. UV-Vis spectra for adsorption of dyes as function of contact time at acidic pH (a) BG (b) MG (c) CR

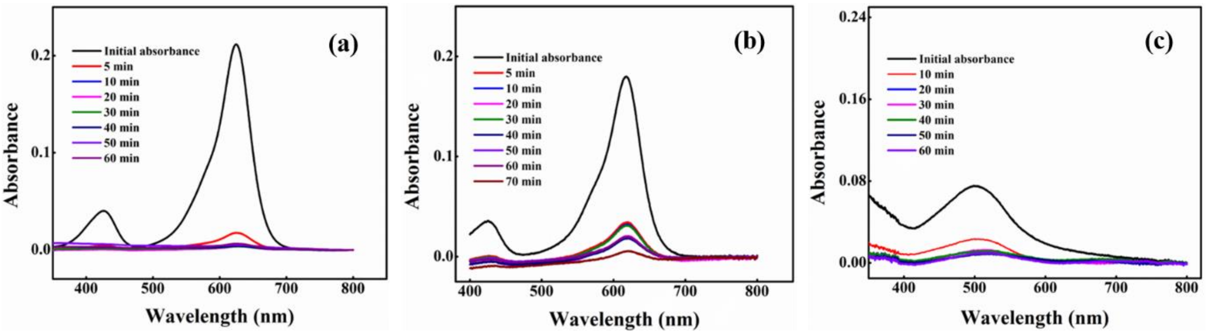

Figure S12. UV-Vis spectra for adsorption of dyes as function of contact time at native pH (a) BG (b) MG (c) CR

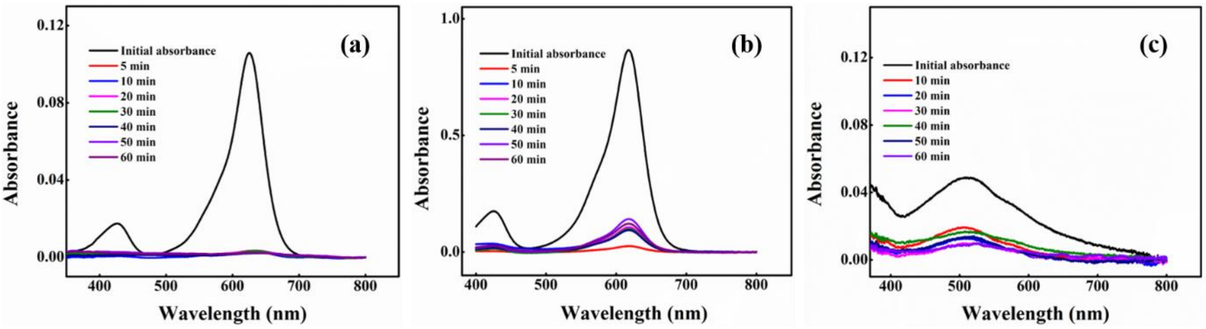

Figure S13. UV-Vis spectra for adsorption of dyes as function of contact time at basic pH (a) BG (b) MG (c) CR
